# A Measurement Approach for Characterizing Temperature-Related Emissivity Variability in High-Emissivity Materials

**DOI:** 10.3390/s25020487

**Published:** 2025-01-16

**Authors:** Gloria Cosoli, Paolo Chiariotti, Beatriz García-Baños, Giuseppe Pandarese, Felipe L. Peñaranda-Foix, Gian Marco Revel

**Affiliations:** 1 Department of Theoretical and Applied Sciences, eCampus University, 22060 Novedrate, Italy; 2 Department of Industrial Engineering and Mathematical Sciences, Università Politecnica delle Marche, 60131 Ancona, Italy; g.pandarese@staff.univpm.it (G.P.); gm.revel@staff.univpm.it (G.M.R.); 3 Department of Mechanical Engineering, Politecnico di Milano, 20156 Milan, Italy; 4 ITACA Institute, Universitat Politècnica de València, 46022 Valencia, Spain; beagarba@upvnet.upv.es (B.G.-B.); fpenaran@dcom.upv.es (F.L.P.-F.)

**Keywords:** emissivity, material characterization, high-emissivity materials, microwave, uncertainty analysis

## Abstract

The effective knowledge of emissivity is pivotal to obtain reliable temperature measurements through non-contact techniques like pyrometry and thermal imaging. This is fundamental in high-temperature applications since material emissivity strongly depends on temperature conditions. Given the recent attention in high-temperature applications, especially for replacing fossil-fuel-dependent heating with greener solutions in energy-intensive processes, renewed interest in characterizing materials radiant properties rose. This work presents a measurement procedure for characterizing the total emissivity of high-emissivity materials exploiting microwaves for heating the test material. The procedure grounds on a sequential approach, using a reference material of known emissivity (e.g., high-emissivity coating, already characterized sample holder, etc.) to derive the target material total emissivity. Uncertainty analysis is performed to provide a metrological characterization of the approach. The procedure is validated on target materials of known emissivity, focusing on high-emissivity materials commonly employed in microwave heating processes. Results are compatible with reference literature and material datasheets, demonstrating the validity of the proposed approach.

## 1. Introduction

The characterization of materials in terms of their radiant properties is pivotal to measure temperature with non-contact techniques. Indeed, many applications take advantage of non-contact infrared methods; industrial furnaces [1] and the aerospace industry [2] represent a few examples of applications targeting quantitative temperature measurements and radiated heat assessment. Microwave (MW)-based heating is an important field of application of infrared (IR) non-contact sensors (e.g., pyrometers), given that traditional sensors can alter the electromagnetic field inside the cavity and modify the thermal distribution of the treated material. In the last decades MW energy has received more and more attention, being considered as clean and efficient in a plethora of applications (e.g., food [3], medicine [4], ceramic industry [5], inorganic materials sintering [6], plasma treatment [7], organic chemistry [8], calcined clays production [9], etc.). The quality of the final product is heavily dependent on the thermal treatment applied as well as the temperature distribution inside the microwave application chamber; hence, the continuous (and possibly real-time) monitoring of temperature can optimize the quality of the product as well as the energy consumption and is also a mitigation method for risks related to MW reactors. Anyway, the development of dedicated monitoring systems is very difficult, especially at high temperature ranges as those needed to produce materials like calcined clays, ceramic pigments, and iron-bearing residues. Non-contact sensors undoubtedly have great potential for these applications, not suffering from the limitations of standard sensors like thermocouples and resistance temperature detectors (RTDs), whose use is limited to approximately 1500 °C, at which there are convective heat losses, and their performance degrades (e.g., because of corrosion phenomena).

The working principle of non-contact temperature measurement systems relies on the Planck’s law, which generalizes into the Stefan–Boltzmann’s law if broadband sensors are considered. Therefore, the materials characteristics—in particular, spectral emissivity—heavily impact on the obtained results (dual-color pyrometers, or ratio-pyrometers, can provide the temperature of a target without the knowledge of emissivity data [10]. Nevertheless, they should be used under specific conditions, such as when the target has a constant emissivity ratio or when partial blocking of the pyrometer’s optical path may occur. Moreover, they are less commonly used than monochromatic or narrow-band pyrometers given their higher cost—typically, 50–100% more than other pyrometers). It is worthy to note that many different conditions must be considered as affecting emissivity, such as heating technologies, spectral band for the measurement (e.g., emittance of metals decreases with increasing wavelengths [11]), type of measurement output, geometry and roughness of the target surface (roughness and surface characteristics heavily influence emissivity [12]—this is the reason, for instance, justifying the attention needed to characterize the emissivity of powder materials [13]), eventual changes in shape, chemical reactions occurring during an industrial process, etc. The surface roughness determines the surface optical response at different wavelengths, hence affecting spectral emissivity. Hence, radiant emissivity can be the main source of uncertainty when dealing with non-contact, high-temperature measurements. Errors in the range of 15–20% on emissivity reflects on errors up to 200 °C in the measured temperature when dealing with high-temperature applications [14]. Therefore, the knowledge of the optical properties of the target object is essential to get an effective estimation of the temperature of a surface. This is crucial with real materials, for which spectral emissivity changes with wavelength and temperature. When the measurement result is exploited for control purposes, it is pivotal to perform an accurate estimation of emissivity. In literature, many groups have worked on this topic. For example, Honner and Honnerová [15] reviewed the radiometric methods to measure emissivity, considering methods of different types. The estimation of emissivity is indeed a tricky process. Both direct and indirect methods can be exploited, consisting in calorimetry/radiometry and reflectivity/transmissivity determination methods, respectively [16]. The use of blackbodies as reference and of vacuum systems is very common to improve the measurement accuracy [17], even if their application is not always straightforward (e.g., oxidation phenomena can occur in vacuum systems). Zhu et al. [18] proposed an instrument for the estimation of the emissivity of metallic materials in the spectral range of 200–450 °C, exploiting black-cup, gold-cup, and dual-cup methods. They achieved an expanded uncertainty (k = 2) < 0.058 in a spectral range of 2.1–2.5 µm. High temperature values are of great interest [19]. Arduini et al. [20] proposed an improved apparatus working until high temperature (i.e., 2400 K) for the accurate measurement of the total directional emissivity, based on an inductive heating unit and the use of a double-walled vacuum vessel. They obtained uncertainty values < 4% for most of the considered spectral range (1–20 µm). The need for measuring emissivity at working temperature was highlighted by Fuente et al. [21], considering a temperature range of 50–1000 °C for solar selective coatings. They made measurement with a handmade radiometer working in the 0.83–25 µm range and operating in a vacuum or in a controlled atmosphere. Different materials require different measurement methods. Vishnevetsky et al. [22] considered opaque low-reflectance materials and developed a method based on active modulation of the background radiation. They exploited an IR camera and an integrating sphere to measure the hemispherical directional emissivity, and they modulated the background radiation through an IR emitter and a mechanical shutter. Also, the measurements should be performed in situ in the environments of interest [23] to obtain results as accurate as possible for the specific application. Indeed, industrial applications pose challenges for temperature measurements. For example, Haggvist et al. [24] proposed a procedure for emissivity estimation for high-temperature assessment on hot titanium alloys, evidencing both industrial constraints and optical changes of the processed material.

Another way to improve the accuracy of temperature measurements consists in exploiting high-emissivity coatings, designed to provide high stability in thermal processes, as blackbody radiators, by applying them to the inner walls of a cavity [25] or even on metal surfaces [26]. Their application inside MW reactors deserves particular attention since the high content of metal powders can alter their behaviour through unexpected chemical reactions inevitably affecting their performance. The knowledge of their emissivity over temperature as provided by manufacturers should be verified in real operating conditions when they are used as reference materials in high accuracy-demanding applications [27]. On the other hand, these paints could be considered as reference materials to be exploited when other materials must be characterized in terms of emissivity. The methodology proposed in this paper could be applied also to these types of products.

It would be important to develop a measurement approach for characterizing emissivity considering the materials typically used in microwave heating processes, such as high-emissivity paints. It would be important to have a method smoothly applicable in industrial contexts so as to improve the measurement quality also for control purposes. Moreover, an uncertainty analysis related to this type of assessment would be beneficial in order to express the results within a precise confidence interval, hence enabling the proper interpretation and exploitation of the data.

The paper is organized as follows: Section 2 will address the materials and methods, while Section 3 will discuss the main results of the activity. Section 4 will report the main conclusion of the work.

## 2. Materials and Methods

The authors propose a sequential measurement procedure for the characterization of materials emissivity of target high-emissivity materials placed inside holders and inserted in an MW reactor, based on the exploitation of a reference material with known emissivity as well as its associated measurement uncertainty. Whatever the form of the material to be characterized (solid, liquid, or powdered), it is fundamental that the material adequately fills the holder and that air voids are avoided/minimized to ensure good contact between the sample and the holder walls, hence ensuring the repeatability and the reproducibility of the measurement. The approach is a two-step one:

1. Characterization of the holder total emissivity over the temperature range of interest: the holder (e.g., a quartz holder of known thickness) is filled with a material whose emissivity (and the associated uncertainty) is known in the temperature range of interest. In this way, the temperature-dependent emissivity of the holder can be identified. It is worthy to note that the uncertainty analysis is fundamental in this context and should be considered part of the proposed estimation approach.
2. Characterization of the target material over the temperature range of interest: the target material is placed in the holder (in a quantity of 1.5 g) and heated in the MW reactor used in the first step. Since the holder emissivity is known from the previous step, the target material emissivity can now be estimated.

It is worthy to note that both steps lead to relevant results, with a procedure providing two cascade emissivity characterizations.

### 2.1. Test Setup

**Figure 1:**
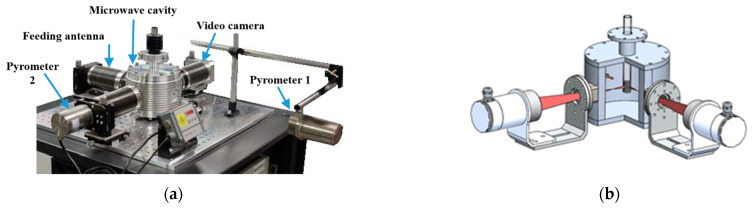
Microwave setup: (**a**) picture; (**b**) section view of the MW reactor with the sample positioning and the beams from the two pyrometers.

Figure 1 shows the microwave setup used in the experiments. It is worthy to note that the positioning of the different components in the setup was fixed and controlled for ease of repeatability.

**Figure 2:**
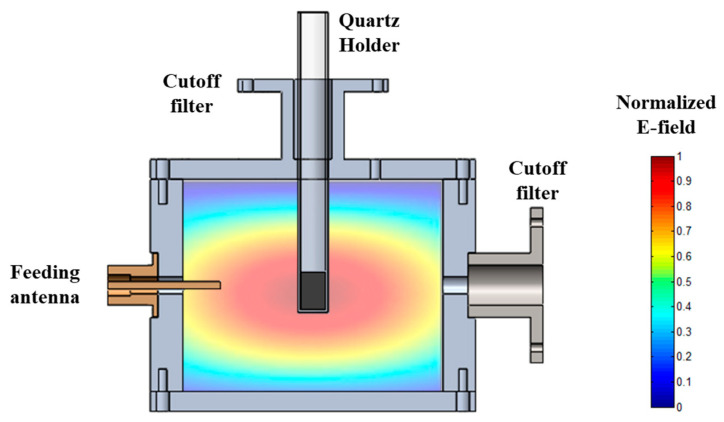
Schematic drawing of the microwave cavity with the sample inside the quartz holder and the distribution of the Electric field with the TE111 mode calculated with the electromagnetic simulator Quickwave-3D (QWED, Poland).

The procedure was carried out in a MW reactor based on a microwave cylindrical cavity (104.92 mm in diameter, 85 mm in height) designed to heat material samples of 10 mm diameter and 15 mm height placed inside a quartz tube container. A feeding antenna couples the TE111 transverse electric microwave mode in the MW reactor around the specific ISM (Industrial, Scientific, and Medical) frequency of 2.45 GHz. Figure 2 presents the normalized distribution of the electric field calculated with the commercial 3D electromagnetic simulator Quickwave (QWED, Warsaw, Poland). As illustrated in the figure, this mode presents a configuration of electric field maximum and uniform in the centre of the cavity, where the sample is placed, ensuring that the material is uniformly irradiated and preventing thermal gradients or the formation of hot spots that could lead to inaccuracies in the temperature determination. The cavity has open accesses in the upper and lateral walls with cutoff filters to avoid microwave leakage to allow the insertion of the quartz tube containing the sample, image recording, and temperature monitoring. Since we are dealing with high-emissivity materials and narrow measurement areas generated by the focal lengths of the lenses of the pyrometers, parasite reflections possibly caused by polished metallic walls can be neglected.

The microwave signal was generated by a Vector Network Analyzer (Rohde & Schwarz, Munich, Germany) and amplified with a 120 W solid-state amplifier (RFcore Ltd., RCA2026U50, Seongnam, Republic of Korea). The control system includes a variable coupling device (that modifies the penetration of the feeding antenna inside the cavity) and a directional coupler (model 722-40, Meca Electronics, Denville, NJ, USA) to measure and adjust the delivered microwave power as a function of frequency and temperature. This system was controlled through a PID ensuring a precise level of microwave power delivered to the sample to maintain the desired heating rate during both heating and cooling cycles. More details about the microwave setup can be found in [28].

A quartz holder of 1 mm wall thickness (model EN09, Vidrasa S.A., Ripollet, Spain) was used as a holder for the materials to be heated. Quartz was chosen as it does not interact with the MW field and just slightly contributes to increase the target material temperature. Moreover, the transmissivity window of this type of quartz is equal to 90% up to 3.5 µm, while drops to 0 for longer wavelengths (according to the specifications provided by the manufacturer). Quartz transmissivity can be considered invariant with temperature, at least in the considered (limited) temperature range.

**Figure 3:**
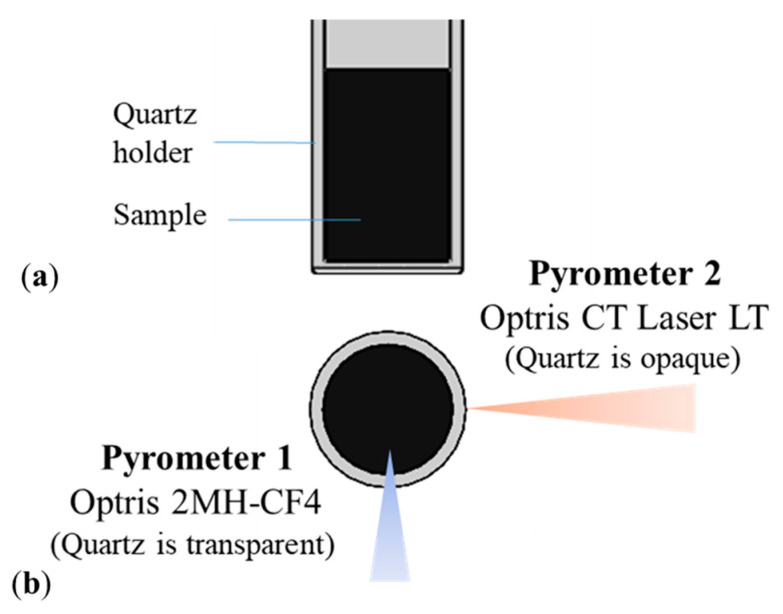
Experimental test setup: material to be characterized in the quartz holder with two pyrometers to which quartz is transparent or opaque, (**a**) top and (**b**) frontal views.

Two pyrometers were used to measure the temperature of both the quartz holder and the sample material (Figure 3), namely:

- Pyrometer 1: Optris 2MH-CF4 (Optris, Berlin, Germany), working at 1.6 µm, for which quartz is transparent. Its spot size is 1.5 mm at 450 mm; furthermore, it has an accuracy equal to ±[0.3%(T_measured_) + 2] °C, a repeatability of ±[0.1%(T_measured_) + 1] °C, and a temperature resolution of 0.1 °C. This pyrometer was used to measure the temperature of the material inside the holder.
- Pyrometer 2: Optris Ct Laser LT, with optics CF2 (Optris, Berlin, Germany), working in the spectral range 7–14 µm, where the employed quartz is opaque. Its spot size is 1.9 mm at 150 mm. Furthermore, the system accuracy is equal to ±1% (or ±1 °C), the repeatability is equal to ±0.5% (or ±0.5 °C), and the temperature resolution is 0.1 °C. This was used to measure the temperature of the quartz itself.

Their metrological characteristics can be considered suitable for the application in the considered temperature range (i.e., up to approximately 750 °C).

For the purpose of heating control, the bulk temperature of the sample was determined from the surface temperature of the holder (measured by pyrometer 2), after a thorough calibration procedure detailed in [29]. The resultant uncertainty in bulk temperature determination was ±4 °C within the temperature range of 20–1000 °C.

### 2.2. Materials

**Figure 4:**
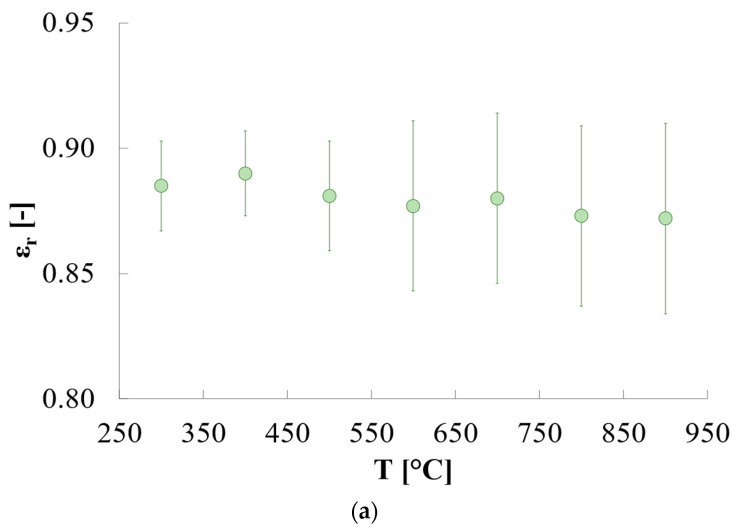

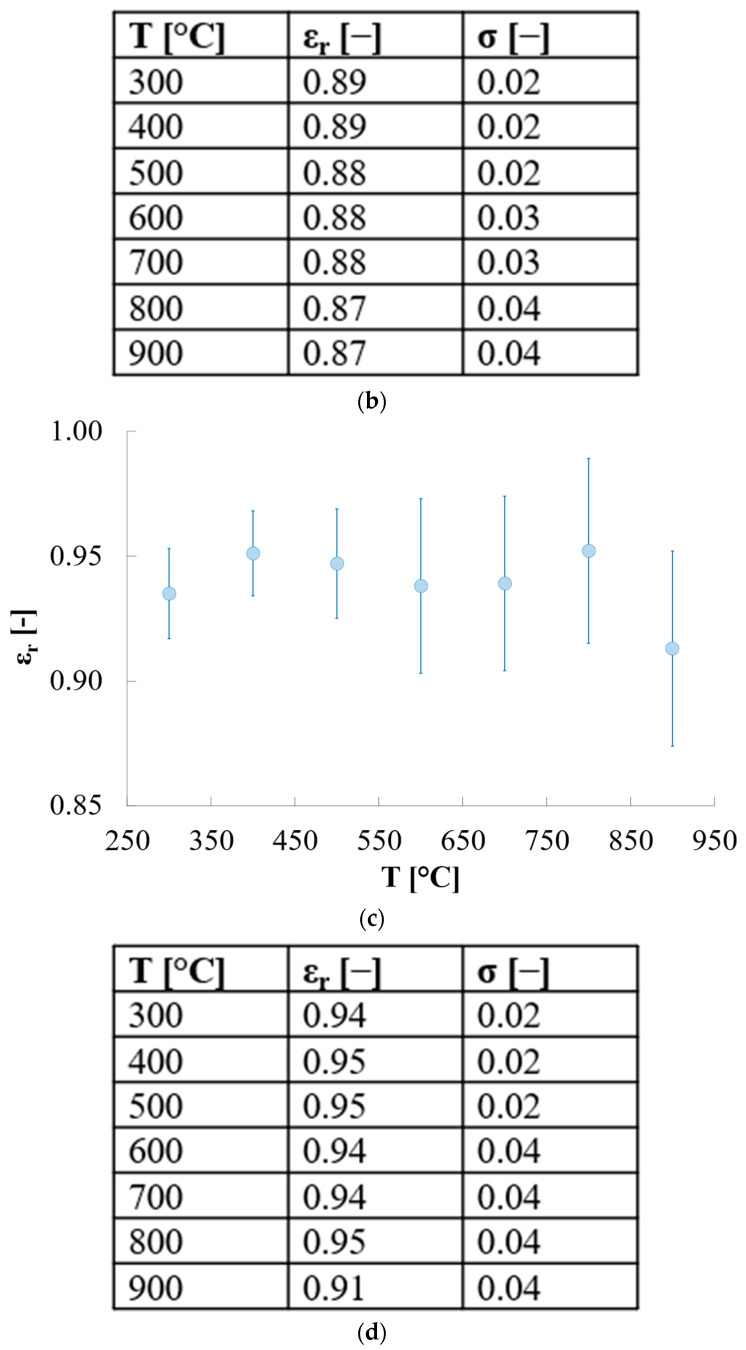
Relative emissivity values of reference paints provided by the manufacturer: (**a**) HiE-Coat 840-C—graphical; (**b**) table format; (**c**) Hie-Coat-840-CM—graphical; (**d**) table format.

The HiE-Coat 840-C paint (AREMCO, Valley Cottage, NY, USA) was used as the reference paint, whereas the paint HIE-Coat 840-CM paint (AREMCO, Valley Cottage, NY, USA) was used for the validation phase. Their emissivity values are provided by the manufacturer for different temperature values together with the associated standard deviation (Figure 4). These coatings are considered as references for emissivity measurements and commonly used in the literature related to emissivity-determination procedures [18]. Prior to measurements, both paints were poured into the quartz holder and cured following the manufacturer procedure. The curing of HiE-Coat 840-C was accomplished by air-drying at room temperature for 1 h, then heating for 1 h at 95 °C. HiE-Coat 840-CM was air-dried at room temperature for 1 h, then heated for 30 min at 95 °C and 1 h at 260 °C.

### 2.3. Test Protocol

Five repeated tests were performed for characterizing the quartz holder for evaluating the repeatability of the process and analysing the variability of the results. Each test consisted of five heating/cooling cycles of the quartz holder filled with the reference paint with controlled slope (i.e., ±10 °C/min), for a total of 25 heating/cooling cycles. This slope was selected to ensure thermal equilibrium between the sample and the quartz holder. Indeed, the MW heating process is rapid, the quartz holder is thin, and the paint mass is limited, so no deviations are expected. The relative emissivity was set at 1 for the acquisitions with both pyrometers.

The emissivity of quartz was derived from data related to both the heating and cooling phases. Consistency of the results obtained from the heating and cooling cycles ensured that there were no chemical reactions that could affect the optical properties of the material during the experiments.

**Figure 5:**
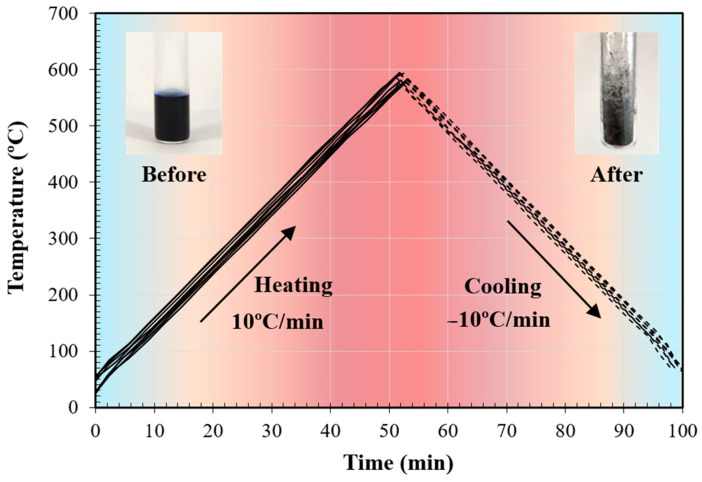
Example of temperature curves recorded during several heating/cooling cycles at rates approx. ±10 °C/min. A picture of the paint sample inside the quartz tube before and after the experiments is presented.

Figure 5 represents typical temperature curves during several heating and cooling cycles (approximate rate: ±10 °C/min), with a picture of the reference paint sample inside the quartz holder before and after a set of experiments. It can be noticed that part of the solvent evaporates after the first heating/cooling cycles, confirming that these thermal processes would slightly affect its characteristics if only a few cycles were performed [27].

Concerning the validation phase, the procedure was tested on a further material, namely another high-emissivity coating, HiE-Coat 840-CM. Since this paint changes its composition for temperatures above 530 °C [27], the test was performed with a heating/cooling cycle up to the maximum of 530 °C and the obtained emissivity was compared with the one provided by the manufacturer, as reported in Figure 4. The aim of this validation phase was twofold: (i) to verify that by using a different reference material the quartz emissivity estimated through the proposed approach is compatible with the results obtained in the first part of the experiment (taking care also of the related measurement uncertainty), and (ii) to characterize the paint emissivity using quartz as reference material, as proposed in the second step of the approach discussed in this paper. These two objectives are reported as step 2 and 3 in Table 1, respectively.

As an additional proof of the validity of the proposed method and to consider a wider temperature range, a further validation step was carried out using the HiE-Coat 840-C paint (step 4 and 5 in Table 1). In particular, the method was applied to two consequent heating/cooling cycles; in the former, the quartz was considered as reference material, whereas in the latter it was the material to be characterized.

For the sake of clarity, the different steps performed in the experimental tests are summarized in Table 1.

### 2.4. Data Processing

The proposed approach grounds on the hypothesis that when two materials, which are monitored through heat radiation-based non-contact approaches, reach thermal equilibrium, the raw data provided by the instruments are related to the temperature as per their radiated power, which is indeed emissivity-dependent.

The Planck’s law integrated over a certain wavelength range provides the hemispherical radiated power per unit area in that wavelength range (1):

(1) $$W_{\Delta \lambda }\left(\Delta \lambda ,T\right)=\int_{\lambda_{1}}^{\lambda_{2}}\frac{\epsilon (\lambda ,\;T)2\pi hc^{2}}{\lambda^{5}\left[e^{\frac{hc}{k\lambda T}}-1\right]}d\lambda$$

where:

- $W_{\Delta \lambda }(\Delta \lambda ,T)$ (W/m^2^) is the radiant exitance (heat flux), i.e., the power emitted per unit area over a Δλ (m) spectral range and for a specific absolute temperature *T* (*K*).
- ϵ(λ, T) is the hemispherical spectral emissivity at a specific spectral wavelength λ (m) and for a specific absolute temperature *T* (*K*).
- c (m/s) is the speed of light in vacuum, equal to approximately 3 × 10^8^ m·s.
- h is the Planck’s constant, equal to approximately 6.6 × 10^−34^ J·s.
- k is the Boltzmann’s constant, equal to approximately 1.4 × 10^−23^ J/K.

The Planck’s law well addresses the radiated power measured by narrow-band pyrometers. For large-band pyrometers (e.g., those working in the spectral range 7–14 µm), the Stefan–Boltzmann law (2) is considered to be a valid equation to be used for relating the radiated power measured by the pyrometer to the temperature of the target body.

(2) $$W=\varepsilon_{r}\sigma T^{4}$$

where:

- *W* (W/m^2^) is the radiant exitance (heat flux), i.e., the power emitted per unit area.
- *T (K)* is the absolute temperature.
- σ is the Stefan–Boltzmann constant, equal to approximately 5.67 × 10^−8^ W/(m^2^K^4^).
- $\varepsilon_{r}$(−) is the relative emissivity, that is what is to be characterized.

The Stefan–Boltzmann law considers total emissivity, i.e., emissivity constant over the temperature and the spectral ranges of interest. Nevertheless, in this work it is assumed that the Stefan–Boltzmann law can be considered valid for both the pyrometers: for the large band one assuming that the measured spectral distribution is representative of the total emitted radiation; for the narrow-band pyrometer (working at 1.6 µm with a tolerance that can be estimated at ±0.2 µm) because it targets a high-emissivity material (i.e., high-emissivity paint, which can be considered similar to a black body). In addition, medium-high temperature values are considered, and the peak emission is in the IR spectral range (Wien’s law). Hence, the Stefan–Boltzmann law is considered a good approximation of the pyrometer transfer function for the two instruments. Consequently, all the calculations reported in the following are based on this hypothesis. It is worthy to note that some assumptions can depend on the specific target materials; hence, prior to applying the methodology on materials with different characteristics, these considerations should be reconsidered also depending on the temperature range addressed.

Equation (2) must be considered both for the reference material (i.e., the paint inside the holder, in this case) and the material to be characterized (i.e., the quartz, in the first step of the procedure). Therefore, we can write Equation (3) for the quartz (i.e., unknown material, to be characterized) and Equation (4) for the paint (i.e., reference material):

(3) $$W_{unknown}=\varepsilon_{r,unknown}\sigma T_{unknown}^{4}$$

(4) $$W_{ref}=\varepsilon_{r,ref}\sigma T_{ref}^{4}$$

where:

- $\varepsilon_{r,unknown}$ is the relative total emissivity of test material (to be characterized—i.e., quartz in the first step of this case).
- $\varepsilon_{r,ref}$ is the relative total emissivity of reference material (i.e., the one provided in the datasheet of the high-emissivity coating, in the specific case).

Given the assumptions made on the pyrometer wavelength dependence, it is worthy to specify that the proposed approach intends to characterize total emissivity. For the sake of simplicity, from here onwards we would refer to “total emissivity” by simply reporting “emissivity”.

Moreover, the spot dimensions are so small that we can consider the targeted cylindric holder as a flat surface.

Given the small thickness of the quartz holder and the small amount of paint mass inside, thermal equilibrium, at which the temperature of both holder ($T_{unknown}$) and inner material ($T_{ref}$) is the same, was supposed as in Equation (5):

(5) $$T_{unknown}=T_{ref}$$

Hence, combining Equation (5) with Equation (2), the relationship reported in Equation (6) was derived:

(6) $$\sqrt[{4}]{\frac{W_{ref}}{\varepsilon_{r,ref}\sigma }}=\sqrt[{4}]{\frac{W_{unknown}}{\varepsilon_{r,unknown}\sigma }}$$

If $\varepsilon_{r,ref}$ is known (Figure 4), $\varepsilon_{r,unknown}$ can be derived as reported in Equation (7):

(7) $$\varepsilon_{r,unknown}=\frac{W_{unknown}}{W_{ref}}\cdot \varepsilon_{r,ref}$$

**Figure 6:**
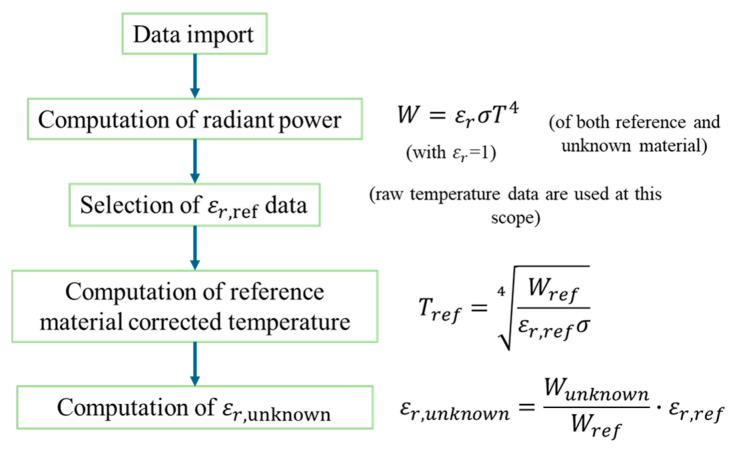
Pipeline of data processing.

The processing pipeline is summarized in Figure 6.

In the first step of the proposed approach, the high-emissivity coating filling the quartz holder was considered as reference materials in all the 25 heating/cooling cycles. Quartz relative emissivity was obtained and compared among the trials. Hence, the average curve of relative emissivity over temperature was derived together with its variability. Hence, measurement uncertainty was estimated.

### 2.5. Monte Carlo Method-Based Simulation for Uncertainty Analysis

To estimate the measurement uncertainty related to quartz emissivity as derived from the approach described above, the Monte Carlo method was exploited to express uncertainty as recommended in the Guide to the Expression of Uncertainty in Measurement (GUM) [30]. This method consists in simulating the process *N* times, where *N* is the number of iterations, considering a certain input uncertainty, and assessing how this value propagates along the whole measurement chain until affecting the output with an estimated value of output uncertainty. Two different analyses were performed in the two phases of the proposed approach (i.e., quartz emissivity estimation and process validation), namely:

- Analysis of measurement uncertainty in the estimation of quartz emissivity, considering as input uncertainty u(x) the value provided by the manufacturer of the reference material (i.e., HiE-Coat 840-C paint)—as reported in Figure 4b). A single trial of a single test was considered to have the data to be perturbed (considering both heating and cooling phases).
- Analysis of measurement uncertainty in the emissivity characterization of a new material (i.e., HiE-Coat 840-CM paint used in the validation part of the study), considering as input the uncertainty estimated in the previous step (which is fundamental in this procedure, as mentioned above, thus should be always considered together with the obtained results so as to properly interpret and use them).

There can be non-controllable experimental factors that could affect the results. The related variability was considered as included in the input uncertainty considered in the MCM-based analysis. A total of 10^6^ iterations were performed for each analysis in order to express the measurement uncertainty as recommended by the GUM [30] (confidence interval of 95%).

## 3. Results and Discussion

The results related to the estimation of quartz emissivity and the associated uncertainty analysis are reported in the first part of this section. Hence, the results from the validation part are described. For the sake of clarity, the step number is reported according to what is described in Table 1.

### 3.1. Estimation of Quartz Emissivity (Step 1)

**Figure 7:**
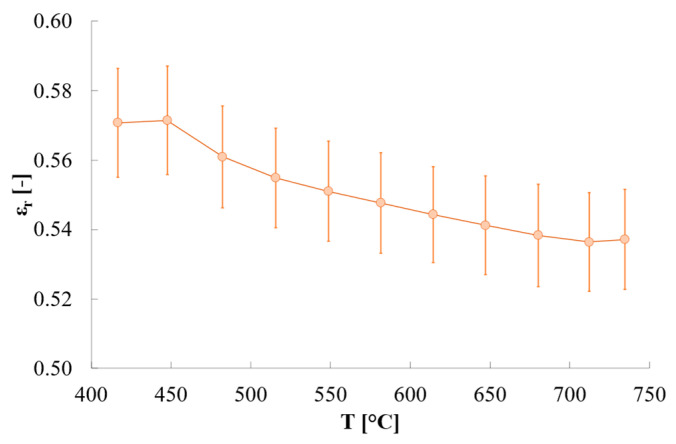
Relative emissivity of quartz reported as averaged value among the 25 heating/cooling cycles. Error bars represent the expanded uncertainty (coverage factor k = 2).

The estimated relative emissivity of quartz is reported in Figure 7. Its variations are quite limited, with values passing from ≈0.57 at ≈450 °C to ≈0.54 at ≈740 °C. The variability among tests is quite low, with standard deviation values < 0.01 whatever temperature is considered.

**Figure 8:**
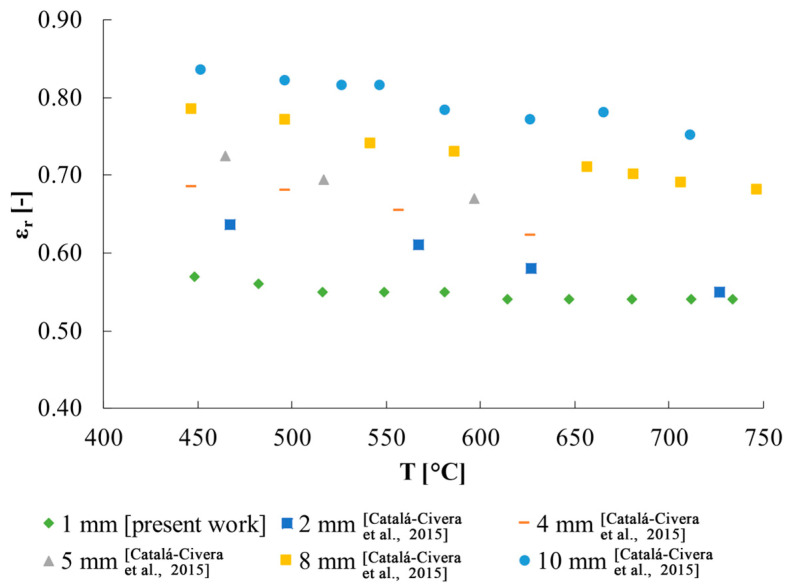
Quartz emissivity: visual comparison of results with literature data [28].

These results are in line with what is expected from literature. In fact, Petrov and Reznik [31] report a quartz relative emissivity of ≈0.64 at ≈700 K (i.e., ≈427 °C), decreasing until 0.41 at 1300 K (i.e., ≈1027 °C) if a thickness of 2 mm is considered. On another hand, with a thickness of 4 mm they obtained a relative emissivity of ≈0.69 at 700 K, decreasing until ≈0.458 at 1300 K. Assuming that the lower the thickness the lower the relative emissivity, the results of the present study are compatible with those reported by Petrov and Reznik (Figure 8). The slightly different slope of the emissivity found with the proposed approach might be due to the different composition of the quartz holder with respect to the quartz samples used by Petrov and Reznik [31].

**Figure 9:**
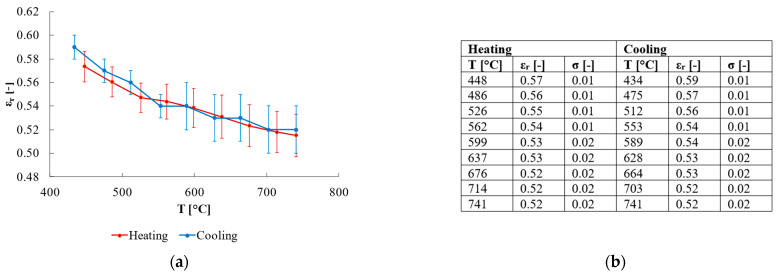
Comparison of quartz emissivity mean values obtained from MCM simulation considering data from heating (red) and cooling (blue) phases. Data are reported both in (**a**) graphical format (expected value ± standard deviation); (**b**) table format.

**Figure 10:**
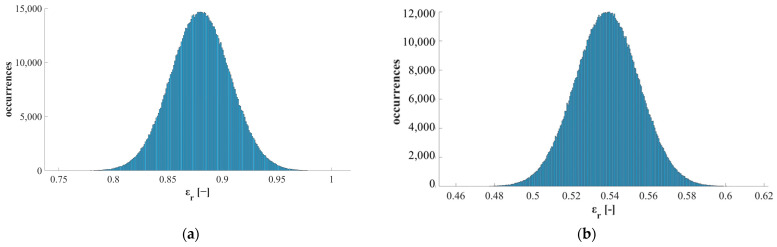
Probability distributions of the relative emissivity: (**a**) HiE-Coat 840-C paint used as reference in the first step of the proposed approach (i.e., input for the estimation of quartz emissivity)—working temperature: 599 °C (heating data considered); (**b**) quartz estimated in the first step of the proposed approach (i.e., output of the measurement pipeline)—working temperature: 599 °C.

Concerning the analysis of measurement uncertainty, the results show that the input uncertainty u(x) related to the reference paint emissivity (in the range of 0.02–0.04 depending on temperature, see Figure 4b) turns into an uncertainty u(y) on the estimated relative emissivity of quartz of ±0.01–0.02 (expanded uncertainty of ±0.02–0.04 with a coverage factor k = 2—values depend on temperature, as reported in Figure 9b)—where temperature is reported as the mean values of corrected temperatures among all the 10^6^ iterations). Compatible results in terms of uncertainty are obtained if data from the cooling phase are considered (Figure 9b)—comparison between mean values obtained is reported in Figure 9a. Examples of the distributions related to the perturbed input (in terms of ε_r_ of the reference paint) and to the corresponding output (i.e., ε_r_ of quartz) are reported in Figure 10a,b, respectively, considering the central value of working temperature (i.e., 599 °C, obtained as average among all the iterations, see Figure 9b). Both the distributions are Gaussian and centred at the expected values of emissivity (Figure 10), namely 0.88 for HiE-Coat 840-C paint and 0.54 for quartz (in agreement with the results obtained for the specific trial considered).

### 3.2. Validation of the Proposed Procedure: Estimation of Quartz Emissivity Considering HiE-Coat 840-CM Paint (Step 2)

**Figure 11:**
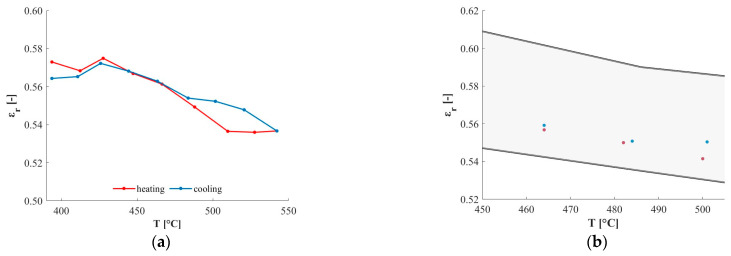
Relative emissivity of quartz: (**a**) values obtained considering HiE-Coat 840-CM paint as reference material (validation phase); (**b**) comparison with MCM-based simulation results (expanded uncertainty interval, with k = 2, is reported).

The first part of validation consisted in estimating the quartz emissivity but now considering the HiE-Coat 840-CM paint as reference material (its datasheet emissivity values are reported in Figure 4d)—step 2 reported in Table 1. The obtained quartz emissivity is reported in Figure 11; it is possible to verify that these values are within the confidence interval obtained with MCM-based simulation (Figure 9b). This further confirms the validity of the proposed approach for emissivity characterization based on a reference material (with known emissivity) and MW heating.

### 3.3. Validation of the Proposed Procedure: Estimation of HiE-Coat 840-CM Paint Considering Quartz Emissivity (Step 3)

In the following step (i.e., step 3 in Table 1), the quartz emissivity data obtained in the first step of the experimental procedure (see previous section) and related measurement uncertainty were used to characterize the second paint (i.e., HiE-Coat 840-CM paint) in terms of emissivity. Because of chemical transitions occurring at high temperature (as proven in [27]) and due to the fact that the paint was not tested as spray coating but used to fill a holder, the test temperature range was limited to 400–500 °C and the results are reported in terms of mean value and standard deviation in this range. Considering 400–500 °C, an emissivity of 0.96 ± 0.02 (k = 2) is obtained from experimental data, whereas the MCM-based simulation provides an emissivity of 0.97 ± 0.02 (k = 2). These results are compatible with the material datasheet, where an emissivity of 0.95 with a standard deviation of 0.02 is reported.

### 3.4. Validation of the Proposed Procedure: Successive Estimation of Quartz and HiE-Coat 840-C Paint (Steps 4 and 5)

**Figure 12:**
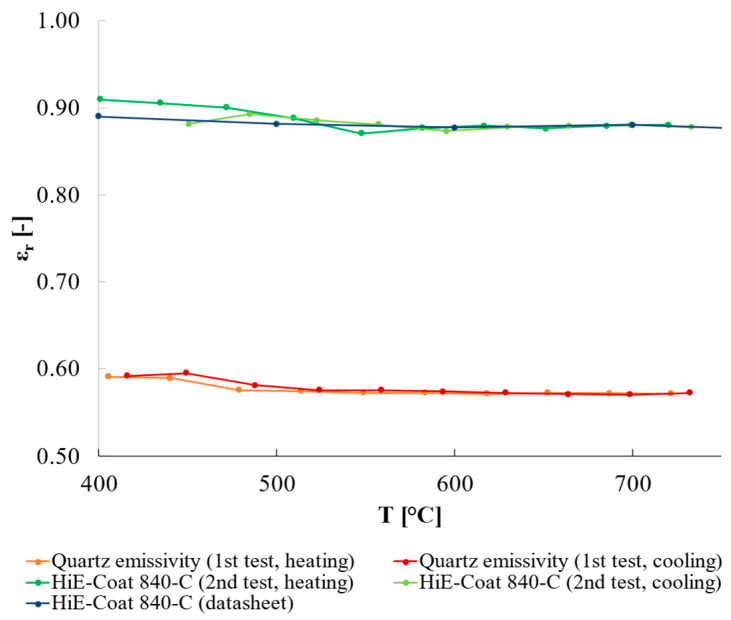
Relative emissivity of quartz (orange and red lines) and HiE-Coat 840-C (dark and light green lines) obtained considering alternatively paint and quartz as reference material in subsequent tests. The blue line represents paint emissivity from its datasheet.

**Figure 13:**
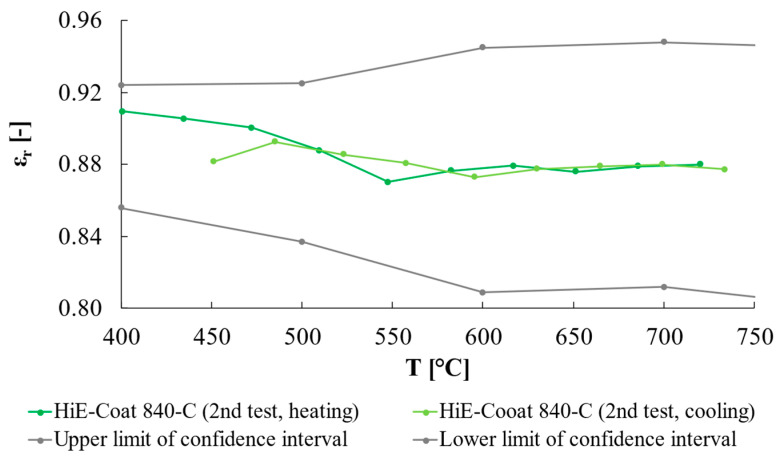
Emissivity of HiE-Coat 840-C estimated considering quartz as reference material. The gray lines represent the confidence interval (k = 2) of the paint emissivity reported in its datasheet.

Concerning the further validation test on HiE-Coat 840-C, the results are reported in Figure 12; the quartz emissivity was estimated from a heating/cooling cycle where the high-emissivity paint (i.e., HiE-Coat 840-C) was used as a reference (i.e., step 4 in Table 1), whereas in the following test, quartz was considered as the reference material and the emissivity of HiE-Coat 840-C was derived consequently (i.e., step 5 in Table 1). As clearly seen in Figure 13 (expanded uncertainty, k = 2, is reported), the results obtained fall in the uncertainty band estimated considering the manufacturer data.

## 4. Conclusions

In this paper, the authors propose a measurement method for characterizing the total emissivity of materials at different temperatures subjected to MW heating using at first a reference material (i.e., a high-emissivity coating) with known emissivity. The attention is focused on high-emissivity materials (with a total relative emissivity > 0.80), given their relevance in the MW field (e.g., high-emissivity coatings). In this way, the need for a reference blackbody is relaxed. Also, the risk of oxidation is reduced because of the faster heating time with MW techniques and, hence, a vacuum system to avoid oxidation phenomena is not needed. The absence of such events makes the results more stable, ensuring a higher level of accuracy. Moreover, a measurement uncertainty analysis is performed to be able to express the measurement results within a certain confidence interval.

The procedure can be summarized as follows:

- The material to be characterized must fill the quartz holder. An MW heating/cooling cycle over the temperature range of interest should be performed, and the temperature of both reference (i.e., quartz) and test materials must be measured with two pyrometers (working in spectral ranges where quartz is opaque and transparent, respectively—dual-color pyrometers were not considered in the specific application, as we could not robustly rely on a-priori constant emissivity ratios).
- The relative total emissivity of the test material should be obtained as an average value between heating and cooling curves to obtain more robust results.
- The measurement uncertainty associated with the resulting total emissivity can be derived considering the expanded measurement uncertainty (with coverage factor k = 2) of quartz provided in this study (i.e., ± 0.01–0.02, depending on temperature, as obtained with the Monte Carlo method) as the input uncertainty of the measurement chain.

The methodology is relatively easy to implement and can be scaled to specific applications in order to consider the specific operating conditions that may affect the emissivity of the material being processed. The uncertainty analysis is part of the proposed approach and should be performed to be able to express the results within a determined confidence interval. It is important to underline that the form of the material under test is fundamental and can represent a limitation to the applicability of the procedure in case that air voids between itself and the holder could not be minimized. Moreover, it is fundamental that the reference material emissivity is provided in function of temperature, so that the characterized emissivity can be detailed in function of temperature (while it is assumed to be constant in the considered spectral range). Furthermore, the conditions to apply the Stefan–Boltzmann law must be fulfilled. It is also worth noticing that the authors are working to extend the results to a wider group of materials involving specimens with different characteristics.

## Figures and Tables

**Table 1:** Summary of the test steps performed.

Test Step	Reference Data	Unknown Emissivity	Objective
1	HiE-Coat 840-C paint	Quartz	Determination of quartz holder emissivity and comparison with literature data.
2	HiE-Coat 840-CM paint	Quartz	Comparison with quartz emissivity obtained in step 1 and check of the consistency of the proposed characterization method.
3	Quartz (whose emissivity was determined in step 1)	HiE-Coat 840-CM	Comparison with manufacturer data, Validation of the proposed method.
4	Quartz	HiE-Coat 840-C paint	Validation in a wider temperature range.
5	HiE-Coat 840-C paint	Quartz	Validation in a wider temperature range, considering as reference data the paint emissivity obtained in step 4.

## Data Availability

Data will be provided upon request to the corresponding authors.
